# Cytotoxic and DNA-Damaging Effects of *Aronia melanocarpa*, *Cornus mas*, and *Chaenomeles superba* Leaf Extracts on the Human Colon Adenocarcinoma Cell Line Caco-2

**DOI:** 10.3390/antiox9111030

**Published:** 2020-10-22

**Authors:** Magdalena Efenberger-Szmechtyk, Adriana Nowak, Agnieszka Nowak

**Affiliations:** 1 Institute of Fermentation Technology and Microbiology, Lodz University of Technology, Wolczanska 171/173, 90-924 Lodz, Poland; agnieszka.nowak@p.lodz.pl; 2 Department of Environmental Biotechnology, Lodz University of Technology, Wolczanska 171/173, 90-924 Lodz, Poland; adriana.nowak@p.lodz.pl

**Keywords:** cytotoxicity, genotoxicity, DNA repair, Caco-2, *Aronia melanocarpa*, *Chaenomeles superba*, *Cornus mas*, polyphenols

## Abstract

*Aronia melanocarpa*, *Cornus mas*, and *Chaenomeles superba* leaf extracts contain large amounts of bioactive compounds—mainly polyphenols, which possess many health benefits including anti-cancer properties. Here, we investigate the biological effects of *A. melanocarpa, C. mas*, and *C. superba* leaf extracts on the human colon adenocarcinoma cell line Caco-2. The antiproliferative activity of the extracts was measured using the MTT assay. The most cytotoxic extract was *C. mas* (IC_50_ = 0.60%). The extracts caused morphological changes in the Caco-2 cells, including partial detachment of cells, necrotic cells, chromatin condensation, cytoplasmic vacuolization, cell nuclei lysis, and nucleus fragmentation. The DNA damage in the Caco-2 cells after exposure to the leaf extracts was measured using the alkaline comet assay. The extracts increased DNA damage in a concentration dependent manner. However, at lower non-cyto- and non-genotoxic (IC_0_) concentrations the extracts induced DNA repair in Caco-2 cells after exposure to hydrogen peroxide. In conclusion, the results of these studies suggest that A*. melanocarpa, C. mas* and *C. superba* leaf extracts can show anticancer activity. However, further research is required on the mechanisms of anti-cancer activity by these extracts, with the application of more advanced and wide-ranging techniques including in vivo experiments.

## 1. Introduction

Plants contain many bioactive compounds, including phenolic compounds such as flavonoids, tannins, and hydroxycinnamic acid derivatives, as well as iridoids, amides, alkaloids, saponins, glycosides, and terpenoids. The most widely investigated phenolic compounds are polyphenols. The best characterized polyphenols are flavonoids, especially catechin, quercetin, and kaempferol derivatives [1]. In recent years, the health benefits of polyphenols have been the subject of increasing research interest. Phenolic compounds are known to offer many health benefits, including anti-diabetic, anti-allergic, anti-atherogenic, anti-hypertensive, anti-thrombotic, cardioprotective, osteoprotective, neuroprotective, anti-aging, hepatoprotective, and anti-cancer effects [2].

According to the World Health Organization (WHO), cancer is the second leading cause of death globally (1 in 6 deaths is due to cancer). In 2018, approximately 9.6 million deaths were caused by cancer. Cancer can affect any part of the body, but the most common types are lung, breast, colorectal, prostate, skin (non-melanoma), and stomach cancers. The development of the disease is related to the rapid creation and growth of abnormal cells, which can invade different parts of the body and spread to other organs. Metastases are a major cause of death. Risk factors associated with cancer include high body mass index, low fruit and vegetable intake, lack of physical activity, tobacco use, and alcohol use [3].

The anticancer activity of polyphenols and plant extracts has been studied extensively. The following mechanisms of anticancer activity by polyphenols have been proposed: prevention of oxidation, detoxification of xenobiotics, inducing apoptosis of cancer cells, stimulating immune system function, estrogenic/anti-estrogenic activity, anti-inflammatory properties, and affecting the cellular Signalling system [4]. Iridoids have also been demonstrated to have potential for chemoprevention and cancer therapy. Other biological properties of iridoids have been reported, such as neuroprotective, anti-inflammatory, immunomodulating, hepatoprotective, antibacterial, antiviral, hypoglycaemic, hypolipidemic, antiallergic, wound healing, anxyolitic, and cardiovascular effects [5].

Polyphenols are present in all parts of plants, including the fruits, leaves, skin, roots, seeds, and flowers. There are many factors that influence the content of polyphenols in plants and plant extracts, such as the plant variety/cultivar, the part of the plant, the growing season, the particle size of the plant material, and the extraction method [6]. Leaves generally have the highest content of phenolics [7,8]. Therefore, leaves are a promising research material and a good source of bioactive compounds. Moreover, they are considered a waste material that can be successfully applied in the food industry. Due to their antioxidant and antibacterial properties, plant extracts rich in polyphenols can be used as natural preservatives for meat and meat products [6,9]. There has also been great interest in the use of plant extracts in functional food products [10].

Several studies have investigated the anticancer activity of plant extracts obtained from leaves. *Micania cordata* leaf extract showed activity against Ehrlich ascites carcinoma (EAC)-induced cancer in mice [11]. Rahman and Akhtar [12] reported the anticancer activity of *Cordia dichotoma* leaves against a human prostate carcinoma cell line (PC3). Gavamukulya et al. [13] demonstrated that *Annona muricata* leaf extracts showed anti-tumour activity against Ehrlich ascites carcinoma cells (EACC) and breast cancer cell lines (MDA and SKBR3).

Previously, we investigated the composition and antibacterial activity of *Aronia melanocarpa, Cornus mas*, and *Chaenomeles superba* leaf extracts against typical meat spoilage and pathogenic bacteria [14]. The extracts were found to possess strong antioxidant properties and to be a rich source of phenolic compounds (mainly phenolic acids and flavonols). Flavones and flavanones were identified in *C. superba*, whereas ellagitannins and iridoids (non-phenolic antioxidant compounds) were found in *C. mas*. All the extracts studied inhibited the growth of food spoilage and pathogenic bacteria, with *C. mas* extract showing the strongest antibacterial properties. There are now plans in place to use *A. melanocarpa, C. mas* and *C. superba* leaf extracts to extend the shelf life and improve the health benefits of meat products.

The aim of the present study was to investigate the biological effects of *A. melanocarpa, C. mas*, and *C. superba* leaf extracts against Caco-2 cells (heterogeneous human epithelial colorectal adenocarcinoma cells). The human colon adenocarcinoma cell line Caco-2 was used as a model cell line. the biological activity of polyphenols depends on their intestinal uptake and metabolism. Most dietary polyphenols occur in plants as glycosylated forms, which are poorly absorbed, favouring their accumulation in the gastrointestinal tract. Intestinal tissue is considered their main site of action [15]. The Caco-2 cell line is used as an in vitro human model of epithelial cells originating from the gastrointestinal tract, which corresponds to the primary target tissue subjected to plant polyphenols in vivo. Caco-2 cells are recognized by the Food and Drug Administration as the most suitable model for intestinal permeability and transport testing, and are widely used for toxicological and pharmacological studies [16]. In these studies, we investigated the cytotoxicity and genotoxicity of *A. melanocarpa, C. mas*, and *C. superba* leaf extracts, and the morphological changes they induced in the Caco-2 cells. Based on the results, we then assessed the ability of chosen concentrations of the plant leaf extracts to induce DNA repair in Caco-2 cells. There have been no previous studies regarding either the cytotoxicity of *C. superba* extracts or the genotoxic activity of *A. melanocarpa, C. mas*, and *C. superba* leaf extracts towards mammalian cell lines. This is also the first study to provide data regarding the effects of these extracts on DNA repair in vitro after the induction of oxidative DNA damage.

## 2. Materials and Methods

### 2.1. Research Material

The research material consisted of the same extracts that were used in the previous studies [14]. *Aronia melanocarpa* (chokeberry), *Chaenomeles superba*, and *Cornus mas* (cornelian cherry) leaves were collected on 15 July 2016 in the village of Tymianka (*A. melanocarpa* and *C. superba*), and in the village of Zadzim (*C. mas*) in central Poland. Bioactive compounds were extracted using water according to the following procedure [14]. 200 g of leaves was mixed with 1000 mL of distilled water. The samples were shaken for 1 h at 4 °C, homogenized for 1 min at 20 °C, filtrated, and centrifuged for 10 min at 10,000× *g* (5804R Centrifuge, Eppendorf, Hamburg, Germany). The supernatant was collected. The extracts were sterilized using microwaves (Microjet, EmbioTechnology, Oensingen, Switzerland), and stored at −20 °C prior to further analysis. The total phenolic content (TPC) of each extract was as follows: 861.6 µg/mL (*A. melanocarpa*); 3110.6 µg/mL (*C. superba*); 1867.7 µg/mL (*C. mas*) [14]. The compositions of the extracts are presented in Table 1.

### 2.2. Chemicals, Reagents, and Culture Vessels

Dulbecco’s Modified Eagle’s Medium (DMEM), HEPES, streptomycin and penicillin, 4′,6-diamidino-2-phenylindole (DAPI), Tris buffer, NaOH, Triton X-100, ethylenedinitrilotetraacetic acid (EDTA), dimethyl sulfoxide (DMSO), thiazolyl blue tetrazolium bromide (MTT), NaCl, Low Melting Point (LMP) agarose, Normal Melting Point (NMP) agarose, Phosphate Buffer Saline (PBS), trypan blue dye, Giemsa stain and May-Grünwald stain were purchased from Sigma-Aldrich, St. Louis, MO, USA. Fetal bovine serum (FBS), GlutaMAX, and TrypLE Express were purchased from Gibco, Thermo Fisher Scientific, Waltham, MA, USA. Roux flasks and a 96-well plates were purchased from Becton, Dickinson and Co., Franklin Lakes, NJ, USA. Lab-TekTM Chamber Slides were purchased from Nunc, Thermo Fisher Scientific, Waltham, MA, USA. Wagner’s reagent was purchased from Chempur (Piekary Śląskie, Poland).

### 2.3. Phytochemical Analysis

The extracts were tested for the presence of active phytochemicals: saponins, alkaloids, steroids, glycosides, carbohydrates, proteins, quinones and coumarine using qualitative tests. The following standard procedures were used [17,18,19].

#### 2.3.1. Test for Saponins

A total of 5 mL of extract with some drops of olive oil was shaken vigorously. The formation of stable foam indicated the presence of saponins.

#### 2.3.2. Test for Alkaloids

A total of 2 mL of the extract was taken for the analysis and 1 mL of hydrochloric acid was added. 2 mL of Wagner’s reagent was added by the side of the test tube. Brown or reddish brown precipitate indicated the presence of alkaloids.

#### 2.3.3. Test for Steroids (Salkowski Test)

A total of 2 mL of the extract was taken for the analysis. 2 mL of chloroform and 2 mL of concentrated sulphuric acid were added to the extract. The formation of dark red colour in lower chloroform layer indicated the presence of steroids.

#### 2.3.4. Test for Glycosides (Keller- Killani Test)

A total of 2 mL of the extract was mixed with 2 mL of glacial acetic acid containing few drops of 2% FeCl_3_. A total of 2 mL of concentrated sulphuric acid was added carefully. A reddish-brown colour in the interphase indicated the presence of glycosides.

#### 2.3.5. Test for Carbohydrates (Fehling Test)

Fehling A and Fehling B reagents were mixed in equal volumes and 2 mL of it was added to 2 mL of the extract. The reaction mixture was boiled gently. The formation of brick red precipitate indicated the presence of reducing sugars.

#### 2.3.6. Test for Proteins

A total of 1 mL of the extract was mixed with 2 mL of Bradford reagent. The formation of blue colour indicated the presence of proteins.

#### 2.3.7. Test for Quinones

A total of 2 mL of concentrated sulphuric acid was added to 2 mL of extract. The formation of red colour indicated the presence of quinones.

#### 2.3.8. Test for Coumarin

A total of 2 mL of 10% NaOH was added to 2 mL of extract. The formation of yellow colour indicated the presence of coumarin.

### 2.4. Caco-2 Cell Culture

The human epithelial colorectal adenocarcinoma cell line (Caco-2) was used. The cells were cultured in Roux flasks as a monolayer in DMEM with the addition of 10% FBS, 4 mM GlutaMAX, 25 mM HEPES, 100 μg/mL streptomycin, and a 100 IU/mL penicillin mixture. The cells were incubated in a CO_2_ incubator at 37 °C in 5% CO_2_ for 7–10 days. The medium was changed every 2–3 days. After reaching confluence, the cells were treated with TrypLE Express for 10 min at 37 °C to detach them, suspended in sterile PBS, then aspirated off the plastic flask. The cell suspension was centrifuged (187× *g*, 5 min) and resuspended in fresh DMEM. The cells were counted using a hemocytometer. Cell viability was determined by trypan blue exclusion. The cells were considered ready to use if viability was at least 90%.

### 2.5. MTT (3-(4,5-dimethylthiazolyl-2)-2,5-diphenyltetrazolium bromide) Assay

The cytotoxicity of the leaf extracts was determined using the MTT assay. Into each well of a 96-well plate was placed 1 × 10^4^ Caco-2 cells and 100 µl of culture medium. The cells were incubated overnight at 37 °C in 5% CO_2_ to allow them to attach. The plant extracts were added to each well at final concentrations of 004; 0.08; 0.16; 0.31; 0.63; 1.25; 2.50; 5.0; 10.00 %. (1:4; *v*/*v*). The negative control contained only DMEM. The cells were incubated at 37 °C in 5% CO_2_ for 72 h. After incubation, the medium was aspirated from each well and 100 µL of MTT (0.5 mg/mL in PBS, pH 7.2) was added. The plates were incubated at 37 °C in 5% CO_2_ for 3 h. The MTT solution was removed and 50 µL of DMSO was added to each well, in order to solubilize the formazan precipitates. Absorbance was measured at 550 nm using a microplate reader (TriStar2 LB 942, Berthold Technologies GmbH and Co. KG, Bad Wildbad, Germany). The reference wavelength was 620 nm. Cell viability (%) was calculated according to the following formula: (sample OD/control OD) × 100%. Cytotoxicity (%) was calculated as 100% − cell viability (%). IC_50_ values were determined according to the OECD protocol [20] as IC_50_ = (X − Z)/(X − X_1_) × (CX_1_ − CX) + CX, where X is a 50% decrease in viability, X is % of viability > Z, X_1_ is % viability < Z, CX is the concentration of the compound for X, and CX_1_ is the concentration of the compound for X_1_.

### 2.6. Microscopic Observations of Morphological Changes

Morphological changes in the Caco-2 cells induced by the plant extracts were observed using four-well Lab-Tek^TM^ Chamber Slides. The concentrations near to or lower than IC_50_ were used: *A. melanocarpa* (2.50%), *C. mas* (0.31% and 0.63%), and *C. superba* (0.63%). The Caco-2 cells were seeded on each well by adding 5 × 10^4^ cells/well. The cells were treated with extract in the same way as for the MTT assay. The cells were incubated at 37 °C in 5% CO_2._ Next, the medium with plant extracts was removed. The cells were rinsed with PBS (pH = 7.2) and fixed with 70% (*v*/*v*) ethanol for 20 min. The ethanol was then removed, and the microscopic preparations were air dried and stained using the May–Grünwald–Giemsa method. Morphological changes in the Caco-2 cells were observed using a phase-contrast microscope (Nikon, Tokyo, Japan) connected to a digital camera (Nikon Digital Sight DS-U3, Tokyo, Japan) and imaging software (NIS-elements BR 3.0, Nikon, Tokyo, Japan) under magnifications of 400× and 1000×.

### 2.7. Genotoxicity Testing (Comet Assay)

The genotoxicity of the plant extracts was determined using the comet assay. The final concentration of Caco-2 cells in each sample was adjusted to 1 × 10^5^. The following concentrations of leaf extracts (near to or lower than the IC_50_) were used: 0.01%, 0.02%, 0.04%, 0.08%, 0.16%, and 0.63%. The comet assay was performed under alkaline conditions (pH > 13), as described previously (Nowak et al., 2017). The cells were incubated with extracts at 37 °C for 1 h. The samples were centrifuged (182× *g*, 15 min, 4 °C). The supernatant was removed and the cells were suspended in 0.75% LMP agarose, layered onto slides pre-coated with 0.5% NMP agarose, and lysed at 4 °C for 1 h in a buffer consisting of 2.5 M NaCl, 1% Triton X-100, 100 mM EDTA, and 10 mM Tris, pH 10. After lysis, electrophoresis was performed in an electrophoretic solution containing 300 mM NaOH and 1mM EDTA to allow the DNA to unwind. Electrophoresis was conducted for 20 min under an electric field strength of 0.73 V/cm (300 mA). The slides were neutralized, air dried overnight and stained with 1 μg/mL DAPI. Comets were observed at 200× magnification using a fluorescence microscope (Nikon Eclipse Ci H600L, Tokyo, Japan) connected to a video camera and a Lucia-Comet v. 7.0 personal computer-based image analysis system (Laboratory Imaging, Prague, Czech Republic). Fifty images were selected randomly for each sample. DNA damage was measured as the percentage of DNA in the comet tails. Two parallel tests with aliquots from the same sample were performed for a total of 100 cells and mean DNA damage was calculated.

### 2.8. DNA Repair

DNA repair was analysed according to the method described by Błasiak et al. [21]. First, the Caco-2 cells were damaged using 25 µM H_2_O_2_ on ice for 10 min. They were then centrifuged (182× *g*, 4 °C, 15 min), resuspended in fresh DMEM, divided into parts and exposed to *A. melanocarpa*, *C. mas*, and *C. superba* extracts at IC_0_ (non-cyto- and non-genotoxic) concentrations of 0.04% and 0.08% for 60 and 120 min at 37 °C. The positive control was exposed to H_2_O_2_ only, while the negative control consisted of cells in DMEM. Aliquots of the suspensions were taken after 60 and 120 min of incubation. To stop the repair activity of the cells, the samples were placed in an ice bath. At each time interval, an alkaline comet assay was performed, as described above. DNA repair was expressed as the extent of residual DNA damage.

### 2.9. Statistical Analysis

Mean values and standard deviations were calculated using Microsoft Excel 2013 software. Analysis of variance (one-way ANOVA) was performed using R 3.4.0 software. Tukey’s Honestly Significant Differences (HSD) test was conducted to determine statistically significant differences between the variables (*p* < 0.05).

## 3. Results

### 3.1. Phytochemical Characteristic of Leaf Extracts

Phytochemical studies revealed the presence of eight groups of bioactive compounds in *A. melanocarpa, C. superba*, and *C. mas* leaf extracts. The following phytochemicals were detected: saponis, alkaloids, steroids, glycosides, carbohydrates, proteins, quinones, and coumarine.

### 3.2. Cytotoxic Activity of Leaf Extracts and Estimation of Half Maximal Inhibitory Concentration (IC_50_)

**Figure 1:**
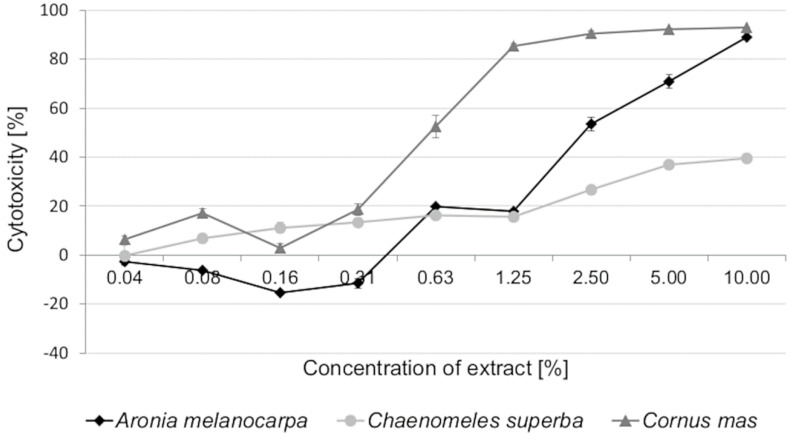
Cytotoxicity of *Aronia melanocarpa, Chaenomeles superba*, and *Cornus mas* leaf extracts in Caco2 cells estimated using the MTT assay after 72 h of exposure. Each data point represents the mean of the absorbance values for cells from four individual wells (±SD).

The leaf extracts decreased the viability of Caco-2 cells in a dose-dependent manner after 72h of exposure, indicating that they had a cytotoxic effect. *C. mas* showed the strongest cytotoxicity (IC_50_ = 0.60%). For *A. melanocarpa* leaf extract, the IC_50_ value was 2.38%. *C. superba* extract had the lowest cytotoxic activity. We were not able to determine the IC_50_ value after 72 h of exposure, because of the low cytotoxic activity. *A. melanocarpa* extract at lower concentrations of 0.04–0.31% increased cell proliferation slightly, suggesting that the cells have a defense mechanism against this plant extract. It was also observed that the cytotoxicity of *C. mas* extract was the highest at concentrations of 2.50–10.00%, reaching 90.46–92.97%. At the highest tested concentration (10.00%), *A. melanocarpa* extract revealed cytotoxic activity of 89.00%, similar to that of *C. mas* (Figure 1).

### 3.3. Changes in the Morphology of Caco-2 Cells

**Figure 2:**
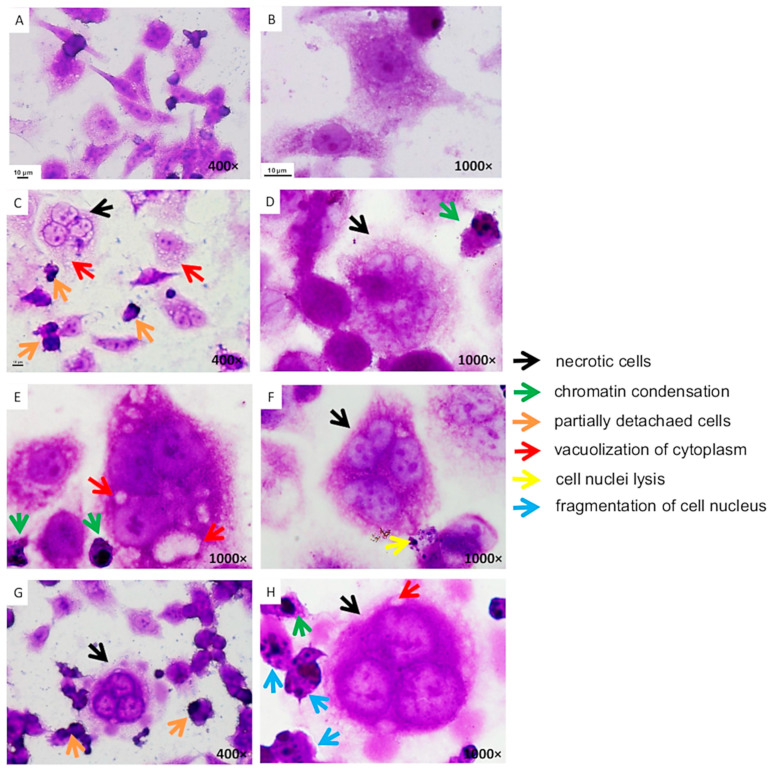
Caco-2 cells stained using the May–Grünwald–Giemsa method after exposure to leaf extracts (**A**,**B**—control cells (not exposed to extracts); **C**,**D**—*Aronia melanocarpa* (2.50%); **E**—*Cornus mas* (0.31%); **F**—Cornus *mas* (0.63%); **G**,**H**—*Chaenomeles superba* (0.63%)).

The Caco-2 cells were exposed to leaf extract concentrations close to or lower than the IC_50_ values. The Caco-2 cells were normally medium-sized with an oval or dendritic shape. They had a clearly defined nucleus and cytoplasm, and formed a regular monolayer. Only a few detached cells were observed in the control sample, which was not exposed to any of the extracts (Figure 2A,B). Significant morphological changes where observed in Caco-2 cells exposed to *A. melanocarpa*, *C. mas*, and *C. superba* extracts. There was a decrease in cell density and destruction of the monolayer, which indicates that the extracts had a cytotoxic effect on the cell membrane. The most common change caused by all the extracts was cell vacuolization (Figure 2C,E,H). This indicates increased metabolic activity (as demonstrated by the MTT test). Frequent detachment of cells from the medium was observed (Figure 2C,G), as well as the presence of necrotic cells (Figure 2C,D,F–H). These morphological changes occurred in large numbers of cells, proving the strong cytotoxic effect of the tested extracts on the intestinal epithelial cancer cell line. Chromatin condensation was noticed in the nucleus of some cells (Figure 2D,E,H). Cell nucleus lysis was observed in cells treated with *C. mas* extract (Figure 2F). *C. superba* extract caused fragmentation of the cell nucleus (Figure 2H). Chromatin condensation and nuclear fragmentation are considered to be symptoms of apoptosis.

### 3.4. Basal and Endogenous DNA Damage Induced by Leaf Extracts

**Figure 3:**
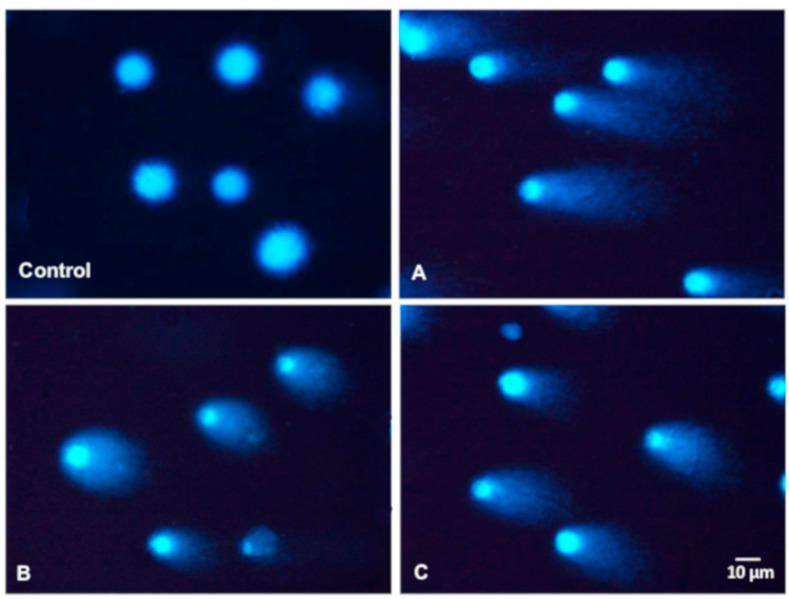
Typical images of DAPI-stained comets exposed to 0.63% leaf extracts: **A**—*Aronia melanocarpa*; **B**—*Cornus mas*, **C**—*Chaenomeles superba*. Magnification 200× (Nikon Eclipse, Japan).

**Figure 4:**
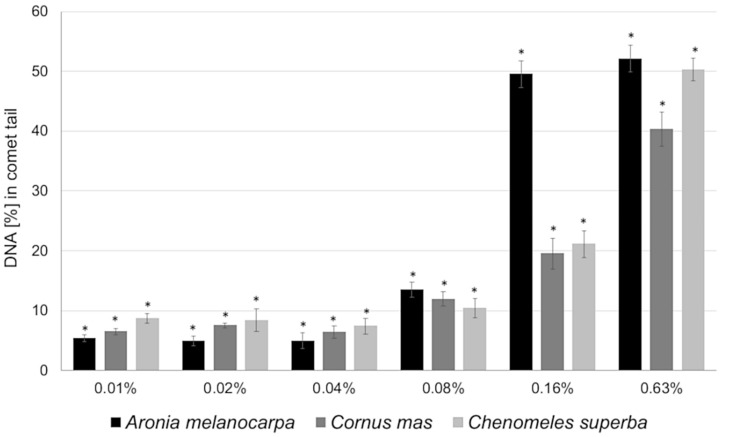
DNA damage in Caco-2 cells after exposure to different concentrations of *Aronia melanocarpa, Chaenomeles superba*, and *Cornus mas* leaf extracts expressed as the mean percentage of DNA in the comet tail according to the alkaline comet assay. Fifty cells were analysed for each treatment. Data from two independent experiments. Error bars denote SEM. (*)—statistically significant differences compared to the control (ANOVA *(p* < 0.05).

**Figure 5:**
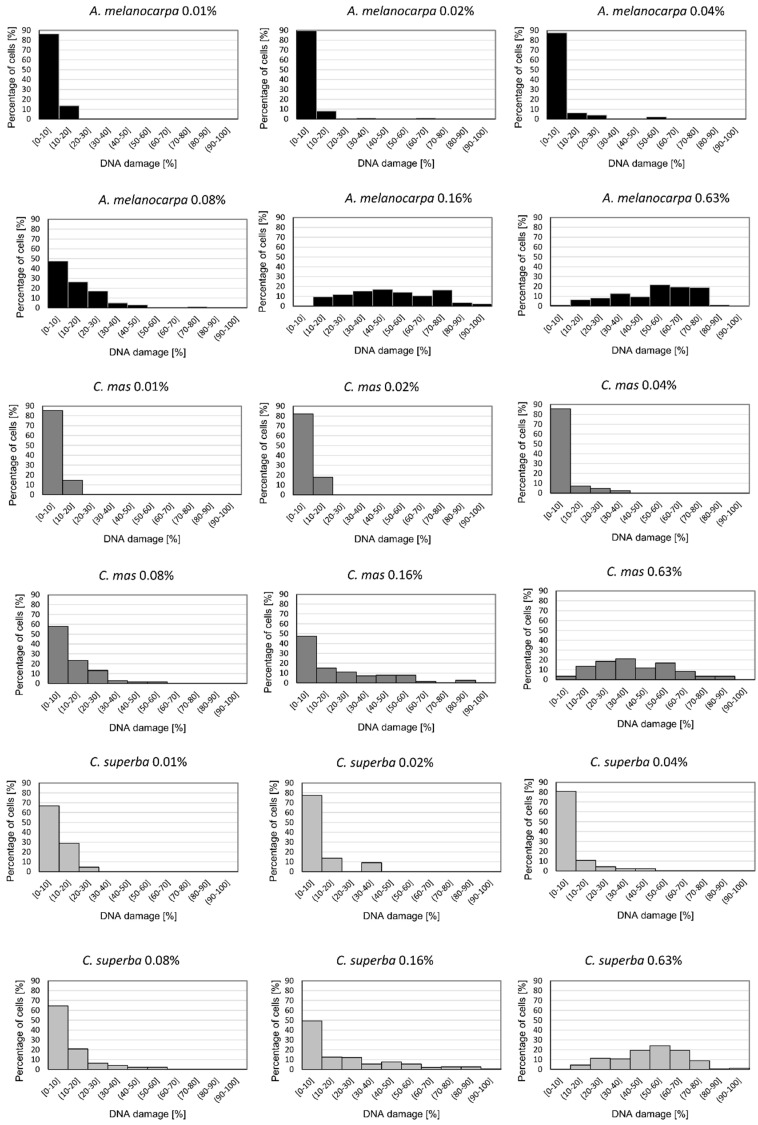
Histograms illustrating the distribution of endogenous DNA damage in Caco-2 cells exposed to *Aronia melanocarpa*, *Cornus mas* and *Chaenomeles superba* leaf extracts at concentrations of 0.01%, 0.02%, 0.04%, 0.08%, 0.16%, and 0.63%.

The comet assay was used to examine the influence of *A. melanocarpa, C. mas*, and *C. superba* extracts on DNA damage in Caco-2 cells. Figure 3 presents typical images of DAPI-stained comets exposed to leaf extracts. The choice of concentrations was based on cytotoxicity testing and IC_50_ values (near to or lower than the IC_50_). Caco-2 cells that were not exposed to extracts (negative control) exhibited DNA damage of 0.93% ± 1.11 (as expressed by the DNA percentage in the comet tail). Plant extracts at concentrations of 0.01%, 0,02%, 0.04%, 0.08%, 0.16%, and 0.63% caused statistically significant DNA damage in the Caco-2 cells compared to the control (*p* < 0.05) (Figure 3 and Figure 4). At higher concentrations (above 2.5%), many apoptotic cells were observed (DNA damage > 80%). No differences in genotoxicity were detected between the extracts at concentrations of 0.01–0.04%. The most potent genotoxicity was observed for *A. melanocarpa* leaf extract (0.16%, 0.63%) and *C. superba* extract (0.63%) (*p* < 0.05) (Figure 4). Figure 5 presents the distribution of Caco-2 cells based on the percentage of damaged DNA in the comet tail. As can be seen, the maximum number of cells with a higher percentage of DNA damage increased with the extract concentration.

### 3.5. DNA Repair in Caco-2 Cells Induced by Leaf Extracts

**Figure 6:**
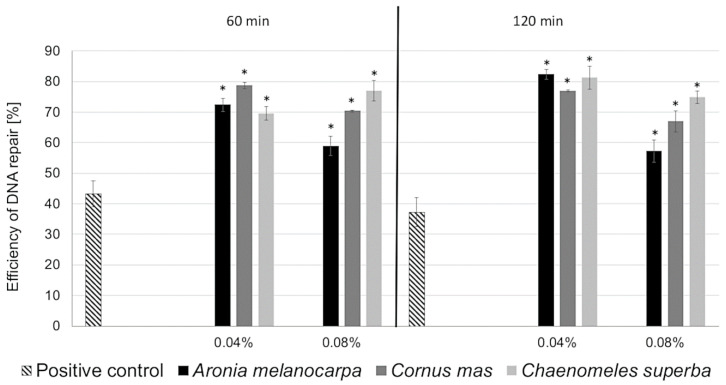
Efficiency of DNA repair in Caco-2 cells exposed to different IC_0_ concentrations (0.04% and 0.08%) of *Aronia melanocarpa, Cornus mas*, and *Chaenomeles superba* leaf extracts. DNA repair was measured at 0 min, after 60 and 120 min of incubation. The number of cells analysed for each time-interval was 50. Error bars denote S.E.M. (*) statistically significant differences from the positive control (ANOVA (*p* < 0.05)).

The *A. melanocarpa*, *C. mas*, and *C. superba* extracts were investigated first for their genotoxic activity. However, at non-cyto- and non-genotoxic concentrations (IC_0_), they can induce DNA repair in Caco-2 cells pretreated with mutagen H_2_O_2_. DNA repair was measured after 0, 60, and 120 min of incubation with the leaf extracts. *A. melanocarpa*, *C. mas*, and *C. superba* reduced the DNA damage in the Caco-2 cells after 60 and 120 min of exposure in a statistically significant manner (*p* < 0.05). For the positive control, the efficiency of DNA repair was approximately 40% after 60 and 120 min of incubation with H_2_O_2_. For all extracts (apart from *C. superba* after 60 min of incubation), the efficiency of DNA repair was greater when a lower concentration was used (i.e., 0.04%). The highest efficiency of DNA repair (>80%) was observed for *A. melanocarpa* and *C. superba* extracts at a concentration of 0.04% after 120 min of incubation (Figure 6).

## 4. Discussion

We used plant extracts obtained from *A. melanocarpa, C. mas*, and *C. superba* leaves in our study. Leaves are generally poorly investigated in the literature. However, they contain more polyphenols than other parts of plants [7,8], which suggest that the extracts obtained from leaves may show stronger biological properties. *Aronia melanocarpa* is commonly known as black chokeberry. Different parts of this plant have been investigated intensively in recent years. Phenolic compounds are probably the most important constituents present in *A. melanocarpa*. The literature data show that *A. melanocarpa* has strong antioxidant potential, probably higher than other plants. Studies have shown the beneficial effects of black chokeberry in common co-morbidities such as obesity, dyslipidaemia, diabetes, hypertension, proinflammatory conditions, and thrombosis risk. Black chokeberry can also have anticancer activity, inhibiting the development of various types of cancers (leukaemia, breast, and intestinal cancers) and cancer stem cells. *A. melanocarpa* can play an important role in treating urinary tract infections, inflammatory bowel diseases, depressive behaviour, and prevent the toxic effects of various substances (e.g., paracetamol) [22,23]. Comparatively little is known about *Cornus mas*, commonly known as cornelian cherry. It contains polyphenols, as well as iridoids—non phenolic compounds with strong radical scavenging potential and anti-tumour properties. *C. mas* has been found to possess antiatherogenic, anti-inflammatory, and neuroprotective effects. The fruits have been used in functional food products such as juices, beverages, jams, yogurts, and vinegar. However, there have been no studies on the applications of leaves and their extracts [24]. *Chaenomeles superba* is a is a hybrid of two species: *Chaenomeles speciosa* and *Chaenomeles japonica* (Japanese quince). Its composition and biological properties have not been studied extensively. To our knowledge, we were the first to investigate the composition of *C. superba* leaves [14]. Different *Chaenomeles* species have been reported to have antioxidant, antimicrobial, and anti-inflammatory activities, as well as antitumor, immunoregulatory, and hepatoprotective effects [25].

In this study, we investigated the influence of plant extracts obtained from *A. melanocarpa*, *C. mas*, and *C. superba* leaves on the human adenocarcinoma cell line Caco-2. In a previous study, we had found that these plants contain high amounts of polyphenols (mainly phenolic acids and flavonols). Flavones and flavanones were identified in *C. superba* leaves, whereas ellagitannins and iridoids were found in *C. mas* leaves [14]. All of these compounds have been reported in the literature to have health benefits, including anticancer properties [26,27]. In this study we performed standard qualitative tests to examine if other active phytochemicals can be present in A*. melanocarpa, C. superba*, and *C. mas* leaf extracts. We showed that the extracts contain saponins, alkaloids, steroids, glycosides, carbohydrates, proteins, quinones, and coumarine. Literature data reveal that all groups of these compounds can be present in leaves [17,18,28]. These compounds were also found to possess cytotoxic activity against cancer cells [29,30,31,32,33,34].

Cytotoxicity resulting in cell death is one of the mechanisms of anticancer activity by polyphenols. These compounds contribute to increase the copper level in cancer cells, exerting a cytotoxic effect by the induction ROS (reactive oxygen species) generation [35]. Cytotoxic chemotherapy uses anti-cancer cytotoxic agents. The cytotoxicity of plant extracts rich in polyphenols and their role in cancer therapy is discussed widely in the literature. In the present study, we have demonstrated the cytotoxicity of *A. melanocarpa*, *C. mas*, and *C. superba* leaf extracts. *C. mas* extract showed the strongest cytotoxic activity, followed by *A. melanocarpa*, and *C. superba* extracts. This suggests that cytotoxicity can be determined by the composition of the extract. *C. mas* leaves contain iridoids, ellagitannins, and caftaric acid, which were not identified in *A. melanocorpa* or *C. superba* leaves [14]. This may explain the stronger cytotoxic effect of *C. mas* extract on Caco-2 cells. The anti-proliferative activity of extracts obtained from *C. mas* flowers, leaves, and fruits has been demonstrated in the literature [36,37,38,39,40], as well as the activity of *A. melanocarpa* leaves, fruits, and stems [41,42,43,44] against cancer cells. To our knowledge, there have been no previous studies on the cytotoxicity of *C. superba* extracts. 

Forman et al. [37] demonstrated that water extracts obtained from the leaves of the *Cornus* L. species *C. mas*, *C. alba*, *C. flaviramea*, *C. kousa*, and *C. officinalis* had a marked cytotoxic effect against human breast cancer cells (MCF-7) after only 24 h. The most effective extracts were from *C. officinalis, C. mas*, and *C. alba*, which decreased the survival of cells by up to 10.3%, 11.1%, and 11.2%, respectively. It was also observed that the anti-proliferative activity of the extracts was positively correlated with the total polyphenol and tannin content. However, high levels of flavonoids and hydroxycinnamic acid derivatives support cells proliferation and protect them from destruction. In our previous studies we identified ellagitannins in *C. mas* leaves [14], which could explain their stronger cytotoxic effect compared to *A. melanocarpa* and *C. superba*. Šavikin et al. [36] demonstrated the cytotoxic activity of extracts obtained from *C. mas* flowers and leaves against human cervix adenocarcinoma HeLa and colorectal adenocarcinoma LS174 cell lines in vitro after 72 h of exposure. The leaf extract was slightly more active than the flower extract against HeLa cells, with IC_50_ values of 60.5 µg/mL and 64.1 µg/mL, respectively. The extracts showed lower cytotoxicity against LS174 cell lines. Against the HeLa cell line, the flower extract (IC_50_ = 131.9 3 µg/mL) was more potent than the leaf extract (IC_50_ = 171.3 µg/mL). In the same study, Šavikin et al. [36] observed no correlation with antioxidant activity, suggesting the mode of action of the tested extracts was not associated with free radical damage.

Yousefi et al. [38] reported the anti-proliferative properties of hydroalcoholic extract obtained from *C. mas* fruits against the following human cancer cell lines after 72 h of exposure: SKOV3 (human ovarian carcinoma); MCF-7 (human breast adenocarcinoma); PC-3 (human prostate adenocarcinoma); and A-549 (lung non-small cell cancer cells). As in our studies, the MTT assay was used. The extract decreased the cell viability of all the cancer cell lines to values below 26%, even at the lowest dose of 5 µg/mL. In all cases, IC_50_ values were below 5 μg/mL. The extract inhibited cells proliferation in SKOV3, MCF-7, PC-3, and A549 cells by 81.8%, 81.9%, 81.6%, and 79.3%, respectively. Tiptiri-Kourpeti et al. [39] demonstrated the cytotoxic activity of *C. mas* fruit juice against four human cancer cell lines and one murine cell line: mammary adenocarcinoma MCF-7, hepatocellular carcinoma HepG2 and colon adenocarcinomas Caco2, HT-29, andmurine colon carcinoma CT26. Cell viability was reduced by 40–50% following incubation with the highest concentration of juice. The juice was most potent against the HepG2 cell line, whereas the MCF-7 cells were the most resistant. Radbeh et al. [40] have reported the cytotoxic activity of free and encapsulated *C. mas* fruit extracts. The extracts showed antiproliferative activity against HT-29cells. The IC_50_ values for the encapsulated extract were 1.33 and 1.47 times higher than those for the free extract, after 24 and 48 h, respectively.

Skupień et al. [41] found that *A. melanocarpa* leaf extract inhibited the growth of the sensitive promyelocytic leukemia cell line HL-60 (IC_50_ = 1.06 g/L) and its multidrug-resistant sublines HL-60/VINC (IC_50_ = 1.325 g/L) and HL-60/DOX (IC_50_ = 1.57 g/L). The *A. melanocarpa* extract was more effective than the mulberry extract, although the phenolic content was lower. Therefore, the content of individual polyphenols and the biochemical interactions between them probably determine the anti-leukaemic activity of these plant extracts. Cvetanović et al. [43] investigated the cytotoxic activity of extracts obtained from leaves and berries of *A. melanocarpa* against the human malignant cell lines HeLa, A-549, and LS174T, as well as normal MRC-5 (human embryonic lung fibroblast). The growth of all malignant cells was inhibited by the extracts. The most sensitive were HeLa cells. The leaf extract exhibited the highest activity, with IC_50_ values of 2.01, 1.38, and 0.69 μg/mL for A-549, LS174T, and HeLa, respectively. The IC_50_ values for normal cells were quite similar to those obtained for malignant cells: IC_50_ = 5.50 μg/mL (berries extract); IC_50_ = 1.72 μg/mL (leaf extract). Gao et al. [44] reported that *A. melanocarpa* fruit extract displays antiproliferative activity, inhibiting the growth of HepG2 human liver cancer cells (IC_50_ = 338.36 µg/mL). It was slightly more effective than blueberry extract and much more effective than haskap berry extract. At concentrations below 350 ug/mL, *A. melanocarpa* extract had no cytotoxic effects. However, cell proliferation was inhibited. This indicates that the inhibition of cell proliferation was not due to cytotoxic activity. Cvetanović et al. [42] studied the cytotoxic activity of extracts obtained from stems of *A. melanocarpa* against the following malignant cell lines: RD (human rhabdomyosarcoma); Hep2c (HeLa derivative); and L2OB (murine fibroblast). The lowest IC_50_ values were observed forL2OB cells. Thani et al. [45] reported that *A. melanocarpa* extract enhanced the cytotoxicity of gemcitamibe chemotherapy medication in the human pancreatic adenocarcinoma cell line ASPC-1.

There have been no previous studies on the effect of *C. superba* extract obtained from any part of the plant on the viability of cancer cells. Chun et al. [46] demonstrated the antiproliferative properties of *Chaenomeles sinensis* fruit extract against HepG2 cells. Kikowska et al. [47] noticed that callus extract of *Chaenomeles japonica* decreased the proliferation of normal skin fibroblasts.

In the present study, *A. melanocarpa, C. mas*, and *C. superba* leaf extracts caused significant morphological changes in Caco-2 cells, indicating a cytotoxic effect. The extracts also caused chromatin condensation and nuclear fragmentation, suggesting cell apoptosis. Necrotic cells also appeared. According to Radbeh et al. [40], the exposure of HT29 cells to *C. mas* fruit extracts also led to segregation of the cell nuclei into fragments, indicating a breakdown in the chromatin that causes DNA condensation. The encapsulated extract increased the rate of DNA fragmentation compared to free extract. Flow cytometry revealed apoptosis and necrosis in HT-29 cells. Kikowska et al. [47] investigated the effect of callus extract of *C. japonica* on themorphology of normal human skin fibroblasts. At a lower concentration (12.5 µg/mL), no differences were observed compared to the control. With a higher concentration of 100 µg/mL, the fibroblasts showed incorrect morphology with narrow projections and many dead cells. According to Nowak et al. [48], ellagitannins of *Rubus ideaus* can lead to chromatin condensation and nucleus fragmentation, which are symptoms of apoptosis. The same authors observed the condensation of nuclear material, apoptotic bodies of different sizes, suggesting late apoptosis, blebbing, and nuclear margination.

Depending on the concentration, polyphenols can act as both antioxidants and pro-oxidants. Due to their antioxidant properties, polyphenols tend to reduce the amount of ROS, protect from DNA damage, or induce DNA repair. Reactive oxygen species (ROS) are one of the main causes of DNA mutation and damage in living cells, and can produce changes in the structure of DNA. They can modify bases, induce inter- and intra-strand crosslinks, promote DNA–protein crosslinks, and create strand break [2,49]. On the other hand, in the presence of some transition ions, such as copper, polyphenols can also act as pro-oxidants, inducing ROS generation which results in DNA degradation [35].

We investigated the influence of *A. melanocarpa*, *C. mas*, and *C. superba* leaf extracts on DNA damage in Caco-2 cells using the comet assay. The extracts increased DNA damage in a concentration dependent manner. *A. melanocarpa* extract was more genotoxic than *C. mas* and *C. superba*, The highest DNA damage was observed at the highest concentrations studied (0.16% and 0.63%). These results suggest that at higher concentrations the extracts act as pro-oxidants, inducing DNA damage. To our knowledge, there is no data in the prior literature regarding the genotoxic activity of *A. melanocarpa, C. mas*, and *C. superba* leaf extracts. However, DNA damage in cancer cells exposed to polyphenols has been described in the literature. Prasad and Katiyar [50] showed that green tea polyphenols had a genotoxic effect on melanoma cells. Nowak et al. [48] described the genotoxicity of ellagitannins obtained from raspberries (*R. idaeus*). A raspberry ellagitannin preparation induced DNA damage ranging from 7.3 ± 1.3% for 2.5 μg/mL to 56.8 ± 4.3% for 80 μg/mL. Individual compounds such as lambertianin C and sanguiin H-6 were less genotoxic, causing DNA damage amounting to 12.3 ± 2.0 (18.9 μM) and 20.4 ± 2.8% (26.7 μM) for lambertianin C and sanguiin H-6, respectively. *C. mas* leaf extract used in our studies was also found to contain ellagitannins [14]. Rocha-Guzmán et al. [51] reported the genotoxic activity of *Quercus resinosa* leaf extracts on HeLa cells. Fresh leaf extract caused more genetic damage than mature leaf extract. Single phenolic compounds, such as catechin and gallic acid, also induced DNA damage. Verschaeve et al. [52] demonstrated the genotoxicity of *Ruscus hypophyllum* fruit and leaf extracts against human C3A hepatic cells. DNA damage was detected at the highest extract concentration studied of 5 mg/mL.

On the other hand, polyphenols also possess protective properties and at appropriate concentrations can induce DNA repair in cells. In the present study, we investigated the ability of *A. melanocarpa, C. mas*, and *C. superba* leaf extracts to induce DNA repair in Caco-2 cells after oxidative damage (exposure to H_2_O_2_). We demonstrated that at lower non-cyto- and non-genotoxic (IC_0_) concentrations (0.04% and 0.08%) the extracts induced DNA repair in Caco-2 cells, acting as antioxidants. A similar effect was observed by Zakłos-Szyda et al. [53], who demonstrated that *Viburnum opulus* fruit phenolic compounds behaved as cytoprotective agents, able to decrease DNA damage in Caco-2 cells at non-cyto- and non-genotoxic (IC_0_) concentrations. According to Tan et al. [54], polyphenols can induce DNA repair by three possible mechanisms: (1) an antioxidative mechanism, by scavenging ROS, decreasing the amount of DNA oxidative damage; (2) enzymatic repair, by promoting the effect of spontaneous enzymatic repair; (3) non-enzymatic repair, by rapid repair of transient DNA damage resulting from ROS attacks before enzymatic repair initiation.

To our knowledge, there have been no previous studies on the effects of *A. melanocarpa, C. mas*, and *C. superba* extracts on DNA repair in eukaryotic cancer cells after oxidative DNA damage. However, the protective properties of other plant extracts rich in polyphenols are well known. Silva et al. [55] reported that co-incubation of HepG2 cells for 4 h with *Gingko biloba* extract (75 µg/mL) and paraquat (1.0 or 1.5 µM) significantly reduced paraquat-induced oxidative DNA damage. Zakłos-Szyda et al. [53] found that phenolic extract from *Viburnum opulus* fruits induced DNA repair after 120 min of pretreatment with H_2_O_2_ or methylnitronitrosoguanidine, reaching 93% and 88%, respectively. Sitarek et al. [56] described the ability of *Leonurus sibiricus* plant extracts to induce DNA repair after H_2_O_2_ treatment and to protect against oxidative DNA damage in Chinese hamster ovary (CHO) cells. Bellion et al. [57] demonstrated the potential of apple extracts obtained from apple juice, pomace, and peels to reduce DNA oxidation damage in Caco-2 cells. After 24 h of incubation, all the extracts significantly reduced menadione-induced DNA strand breaks, predominantly at low concentrations (1–10 μg/mL). Peel extract was the most effective, preventing DNA damage with a maximum reduction of 46%, followed by the pomace extract (53%), and apple juice extracts (50 and 72%). The extracts also reduced cellular ROS level. Peel extract was the most effective, probably due to large amount of quercetin glycosides (16.3%) which were found to exhibit strong ROS-reducing potential [58].

## 5. Conclusions

In conclusion, this study has shown that *A. melanocarpa, C. mas*, and *C. superba* leaf extracts reveal cytotoxic activity inhibiting proliferation of Caco-2 cells. The most potent cytotoxic activity was shown by *C. mas* extract. Morphological changes were also observed in in the Caco-2 cells exposed to *A. melanocarpa, C. mas*, and *C. superba* leaf extracts. Due to their antioxidant properties, at lower concentrations the extracts stimulated DNA repair in Caco-2 cells, whereas at higher concentrations the extracts revealed pro-oxidant activity, causing DNA damage. *A. melanocarpa, C. mas*, and *C. superba* leaf extracts were found to possess strong biological activity towards Caco-2 cells. These properties are related to the high content of flavonoids and phenolic acids in the plant extracts, the presence of ellagitannins and iridoids in *C. mas*, as well as the presence of other bioactive components such as saponins, alkaloids, steroids, glycosides, carbohydrates, proteins, quinones, and coumarine. However, this is a preliminary study on biological effects of *A. melanocarpa, C. mas*, and *C. superba* leaf extracts against Caco-2 and application of more advanced and wide-ranging techniques including in vivo experiments are needed. As future perspective, further research is required on the molecular mechanism/s of anti-cancer activity and the investigation on the possible compound/s responsible for the observed effects have to be made.

## Figures and Tables

**Table 1:** Composition of *Aronia melanocarpa*, *Chaenomeles superba*, and *Cornus mas* leaf extracts [14].

Group of Compounds	*Aronia melanocarpa*	*Chaenomeles superba*	*Cornus mas*
Phenolic acids	neochlorogenic acid, chlorogenic acid, caffeoyl-deoxyhexose, *p*-coumaroylquinic acid	neochlorogenic acid, caffeic acid dimer/caffeoyl hexoside, chlorogenic acid, *p*-coumaroylhexoside, *p*-coumaroylquinic acid isomers	gallic acid, caftaric acid isomers, *p*-coumaroylhexoside
Concentration	114.66 ± 6.50 µg/mL	644.16 ± 35.33 µg/mL	145.1 ± 10.01 µg/mL
Flavonols	quercetin-3-*O*-rhamnoside, quercetin-pentoside-deoxydihexoside, quercetin-3-*O*-dihexoside, quercetin-3-*O*-dirhamnosylhexoside, quercetin-3-*O*-vicianoside, quercetin-3-*O*-robinobioside, quercetin-3-*O*-rutinoside, quercetin-3-*O*-glucoside, isorhamnetin-3-*O*-rutinoside, isorhamnetin-hexoside-pentoside, kaempferol-3-*O*-rutinoside	dihydroquercetin-hexoside, quercetin-3-*O*-rutinoside, quercetin-3-*O*-galactoside, quercetin-3-*O*-glucoside, kaempferol-3-*O*-hexoside, kaempferol-3-*O*-rutinoside, kaempferol-hexosidedeoxyhexoside	quercetin-3-*O* glucuronylpentoside, quercetin-3-*O*-rutinoside, quercetin-3-*O*-glucuronide, quercetin-3-*O*- glucoside, kaempferol-3-*O*-glucuronide
Concentration	93.40 ± 5.66 µg/mL	252.17 ± 13.27 µg/mL	111.66 ± 7.19 µg/mL
Flavones		luteolin-3-*O*-rutinoside, luteolindihexoside	
Concentration	-	17.28 ± 0.86 µg/mL	-
Flavanones		naringenin-7-*O*-hexoside	
Concentration	-	227.30 ± 11.37 µg/mL	-
Ellagitannins			camptothin A isomers, cornusiin F 1 and 2, cornusiin A 1 and 2
Concentration	-	-	151.63 ± 8.77 µg/mL
Ellagic acid	-	-	ellagic acid
Concentration	-	-	2.56 ± 0.27 µg/mL
Substituted phenols	hydroxytyrosol	hydroxytyrosol	
Concentration	7.92 ± 0.40 µg/mL	75.00 ± 5.25 µg/mL	-
Iridoids	-	-	loganic acid isomers, secoxyloganin, cornuside
Concentration	-	-	33.9 ± 1.85 µg/mL
